# Rapid Induction of COOLing in Stroke Patients (iCOOL1): a randomised pilot study comparing cold infusions with nasopharyngeal cooling

**DOI:** 10.1186/s13054-014-0582-1

**Published:** 2014-10-27

**Authors:** Sven Poli, Jan Purrucker, Miriam Priglinger, Matthias Ebner, Marek Sykora, Jennifer Diedler, Cem Bulut, Erik Popp, André Rupp, Christian Hametner

**Affiliations:** Department of Neurology, Heidelberg University, INF 400, 69120 Heidelberg, Germany; Department of Neurology & Stroke, Tuebingen University, Hoppe-Seyler-Str. 3, 72076 Tuebingen, Germany; Department of ENT, Heidelberg University, INF 400, 69120 Heidelberg, Germany; Department of Anaesthesiology, Heidelberg University, INF 110, 69120 Heidelberg, Germany

## Abstract

**Introduction:**

Induction methods for therapeutic cooling are under investigated. We compared the effectiveness and safety of cold infusions (CI) and nasopharyngeal cooling (NPC) for cooling induction in stroke patients.

**Methods:**

A prospective, open-label, randomised (1:1), single-centre pilot trial with partially blinded safety endpoint assessment was conducted at the neurointensive care unit of Heidelberg University. Intubated stroke patients with an indication for therapeutic cooling and an intracranial pressure (ICP)/temperature brain probe were randomly assigned to CI (4°C, 2L at 4L/h) or NPC (60L/min for 1 h). Previous data suggested a maximum decrease of tympanic temperature for CI (2.1L within 35 min) after 52 min. Therefore the study period was 1 hour (15 min subperiods I-IV). The brain temperature course was the primary endpoint. Secondary measures included continuous monitoring of neurovital parameters and extracerebral temperatures. Statistical analysis based on repeated-measures analysis of variance.

**Results:**

Of 221 patients screened, 20 were randomized within 5 months. Infusion time of 2L CI was 33 ± 4 min in 10 patients and 10 patients received NPC for 60 min. During active treatment (first 30 min), brain temperature decreased faster with CI than during NPC (I: −0.31 ± 0.2 versus −0.12 ± 0.1°C, *P* = 0.008; II: −1.0 ± 0.3 versus −0.49 ± 0.3°C, *P* = 0.001). In the CI-group, after the infusion was finished, the intervention no longer decreased brain temperature, which increased after 3.5 ± 3.3 min. Oesophageal temperature correlated best with brain temperature during CI and NPC. Tympanic temperature reacted similarly to relative changes of brain temperature during CI, but absolute values slightly differed. CI provoked three severe adverse events during subperiods II-IV (two systolic arterial pressure (SAP), one shivering) compared with four in the NPC-group, all during subperiod I (three SAP, one ICP). Classified as possibly intervention-related, two cases of ventilator failure occurred during NPC.

**Conclusions:**

In intubated stroke patients, brain cooling is faster during CI than during NPC. Importantly, contrary to previous expectations, brain cooling stopped soon after CI cessation. Oesophageal but neither bladder nor rectal temperature is suited as surrogate for brain temperature during CI and NPC. Several severe adverse events in CI and in NPC demand further studying of safety.

**Trial registration:**

ClinicalTrials.gov NCT01573117. Registered 31 March 2012

**Electronic supplementary material:**

The online version of this article (doi:10.1186/s13054-014-0582-1) contains supplementary material, which is available to authorized users.

## Introduction

Experimental data suggest that compared with late treatment initiation, early commencement of cooling delivers superior neuroprotection in cardiac arrest and stroke [1,2]. Several methods for induction of therapeutic cooling are available [3], but these methods are underinvestigated with regard to their effects on the brain and neurovital parameters [4]. The present study focused on intravenous cold infusions (CI) and the RhinoChill device for nasopharyngeal cooling (NPC; BeneChill, Inc., San Diego, CA, USA).

CI represent the current clinical standard for hypothermia induction in cardiac arrest [5-7] and are presently being used for induction of hypothermia in two large trials investigating the outcome of stroke [8,9]. However, no evidence exists on the efficacy of CI with regard to brain temperature – the primary target for neuroprotection [10]. Measuring tympanic temperature in stroke patients, previous data of CI (2.1 L within 35 minutes) showed a maximum temperature decrease, in mean, of 1.6°C after 52 minutes [11]. Data on the safety of CI in stroke patients are also urgently needed. The usefulness of four observational studies (n = 3 to 36) [11-14] and one small randomised controlled trial (n = 10) [15] is limited by their intermittent rather than continuous circulatory measurements when administering CI, and no data are available on neuromonitoring. Furthermore, a cardiac arrest trial recently found that early hypothermia induction with CI improved neither the survival nor the neurological status of the patients and was associated with an increased rate of pulmonary oedema [16].

NPC is a new method to induce cooling. Both Castrén and Busch with their respective colleagues demonstrated the feasibility and safety of NPC in the prehospital cardiac arrest setting [17,18], and Abou-Chebl *et al*. provided data on brain cooling in brain-injured patients (−1.4°C within one hour) [19]. We confirmed this cooling rate in stroke patients (−1.2°C after 58 minutes), but highlighted critical rises in blood pressure (BP) as a potentially important adverse effect (AE), warranting further investigation [20].

The Rapid Induction of COOLing in Stroke Patients trial (iCOOL1) is investigating, for the first time, the effect of CI on brain temperature and comparing CI and NPC in their brain cooling efficacy and safety during cooling induction. Secondary measures comprise a safety evaluation, including continuous monitoring of neurovital parameters, and the analysis of temperature dynamics at different extracerebral monitoring sites to find a valid surrogate for brain temperature during cooling induction with either CI or NPC.

## Materials and methods

### Study design, approval

iCOOL1 is a prospective, interventional, open-label, two-armed, randomised, single-centre pilot trial with partially blinded safety endpoint assessment (see Randomisation and blinding). Clinical Trial Registration Information [21] unique identifier is NCT01573117. Institutional Review Board approval was obtained from the ethics committee of Heidelberg University (protocol no. S-301/2010). Informed consent was obtained from patients (whenever competent) or their legal representatives before enrolment in the study.

### Setting and eligibility criteria

The study was conducted at the neurointensive care unit (NICU) of the Department of Neurology at Heidelberg University, a tertiary care hospital including a neurovascular centre.

Our NICU has 12 ventilator-equipped beds and more than 200 severe stroke patients are treated per year. Between June and November 2011 all admitted patients were screened and, if eligible, enrolled prospectively. The inclusion and exclusion criteria are listed in Table 1.

**Table 1:** **Inclusion/exclusion criteria**

**Inclusion criteria:**	**Exclusion criteria:**
○ Ischaemic or haemorrhagic stroke	○ Severe cardiac insufficiency (NYHA ≥ III)
○ ICP/temperature brain probe	○ Acute myocardial infarction or pulmonary embolism
○ Indication for therapeutic cooling independent of the study	○ Threatened ventricular dysrhythmia or cardiac dysrhythmia (heart rate <50/min, QTc >450 ms, sick sinus syndrome, AV block II-III°)
○ Sedation, intubation, and mechanical ventilation	○ Chronic sinusitis or current or past fracture or surgery of the paranasal sinuses not allowing secure application of nasopharyngeal cooling
○ Age ≥18 years	○ Severe infection with bacteraemia or sepsis ≤72 h
○ Written informed consent by patient or legal representative	○ Fever >38.5°C
	○ Severe renal or liver insufficiency
	○ Known haematologic disease with increased risk of thrombosis (e.g., cryoglobulinaemia, cold agglutinins or sickle cell anaemia)
	○ Known vasospastic vascular disorder (e.g., Raynaud phenomenon or thromboangiitis obliterans)
	○ Body weight >120 kg

In all patients, neurological intensive care physicians had prescribed therapeutic cooling and placement of a combined intracranial pressure (ICP)/temperature brain probe independent of the study (Neurovent Temp or Neurovent PTO, accuracy ± 0.1°C; Raumedic, Muenchberg, Germany; inserted >3 cm below the cortical surface). According to our standard operating procedure, therapeutic cooling targeting normothermia is indicated when body core temperature rises above 37.2°C [22]. Therapeutic hypothermia as a compassionate measure for treatment of cerebral oedema has been implemented at our institution since 1998 [23], even though the treatment is experimental. All patients were intubated and deeply sedated and showed no response to painful stimuli, including pinching of the nasal septum (RASS-5, Richmond Agitation Sedation Scale). Our standard regimen for sedation and analgesia included the combination of either midazolam and sufentanil or propofol and remifentanil. Pressure-controlled continuous mandatory ventilation (pc-CMV) was applied (SERVO-s/-I; Maquet, Rastatt, Germany) targeting for physiological levels of arterial blood gases (PaO_2_ 80 to 110 mmHg, PaCO_2_ 35 to 45 mmHg).

### Interventions

Patients were randomised to CI (intervention A) or NPC (intervention B).

*Intervention A:* Two litres of cold (4°C) isotonic saline solution (0.9% NaCl) were infused intravenously via a cubital vein catheter (18 gauge or larger) at a constant flow rate of 4 L/hour using a Power Infuser (ZOLL, Sunnyvale, CA, USA). We chose the same volume and infusion rate as employed in most cardiac arrest trials [5,16] and implemented in previous and current stroke trials [9,11-15,24].

*Intervention B:* NPC has been described in detail by others [17,18]. Briefly, NPC uses nasal catheters to spray an inert coolant (perfluorohexane) at a high flow rate into the nasal cavity. A lidocaine spray was applied before introduction of the nasal catheters. At treatment initiation, the flow rate was gradually increased over a period of 2 minutes until a rate of 60 L/minute was reached. NPC was applied for one hour.

During both interventions (A, B) no other cooling procedures were allowed.

### Outcome

Because previous data suggested a maximum decrease of tympanic temperature for CI (2.1 L within 35 minutes) after 52 minutes, a study period of one-hour was chosen. The primary endpoint was defined as the change in brain temperature within one hour after treatment initiation. For better assessment of dynamic temperature changes, the one-hour study period was divided into four predefined subperiods of 15 minutes duration (I to IV). Secondary outcome measures were changes in body temperature at different extracerebral monitoring sites (tympanum, bladder, rectum and oesophagus), neurovital parameters (arterial pressure, cerebral perfusion pressure [CPP], heart rate [HR], ICP and oxygen saturation [SpO_2_]) and ventilation parameters during the one-hour study period, and the assessment of various safety parameters, such as shivering, blood and blood gas analyses, bleeding complications, signs of acute heart failure, local irritations in the nasopharynx and olfactory dysfunction up to six months after cooling.

Critical values of neurovital parameters counted as severe AE and were predefined according to our local guidelines as follows: systolic arterial pressure (SAP) >180 mmHg in ischaemic stroke and >160 mmHg in haemorrhagic stroke; ICP >20 mmHg; CPP <60 mmHg. Relevant delta values were defined as changes in SAP > +20 mmHg and ICP > +10 mmHg. Shivering was graded using the bedside shivering assessment scale (BSAS, ranging from 0 = no shivering to 3 = severe shivering) [25]. BSAS scores of 3 were classified as severe AE. Blood samples for blood gas analyses and blood analysis were drawn before and two hours and twelve hours after the start of cooling [see Additional file 1]. Cranial CT before and after cooling induction and echocardiography were part of the routine diagnostic assessment and the images obtained were evaluated by neuroradiologists and cardiologists blinded for treatment allocation. Three days after cooling induction, patients underwent otorhinolaryngological (ORL) examination, including anterior and posterior rhinoscopy. ORL examination was repeated ≥6 months after the intervention, adding a standardised 16-item smell test with Sniffin’ Sticks. The ORL physicians were also blinded for treatment allocation.

Information about the medical equipment used can be found in the supplementary methods section [Additional file 2].

### Randomisation and blinding

An independent physician randomly allocated groups, marked non-transparent envelopes sequentially, and sealed them. S.P. and C.H. obtained the patient consents and opened the envelopes in predefined order. Patients were randomised at a 1:1 ratio to either of the two interventions: (A) CI or (B) NPC. All neuroradiologists, cardiologists and ORL physicians who examined the patients were blinded to group allocation.

### Statistics

Statistical analysis was performed using MATLAB v7.12 (MathWorks, Natick, MA, USA) and SAS v9.2 (SAS, Cary, NC, USA). The duration of baseline reading (15 minutes), the division of the one-hour interventional period into four subperiods (I to IV, 15 minutes each) and statistical analyses were predefined in the study protocol. Repeated-measurements analysis of variance (ANOVA) with the between-subject effect GROUP (CI versus NPC) and the within-subject effect TIME (five measurements: baseline, interventional periods I to IV) was used for the main statistical analysis (correction according to Greenhouse and Geisser). Further information is given in the supplementary statistics section [see Additional file 2].

## Results

**Figure 1:**
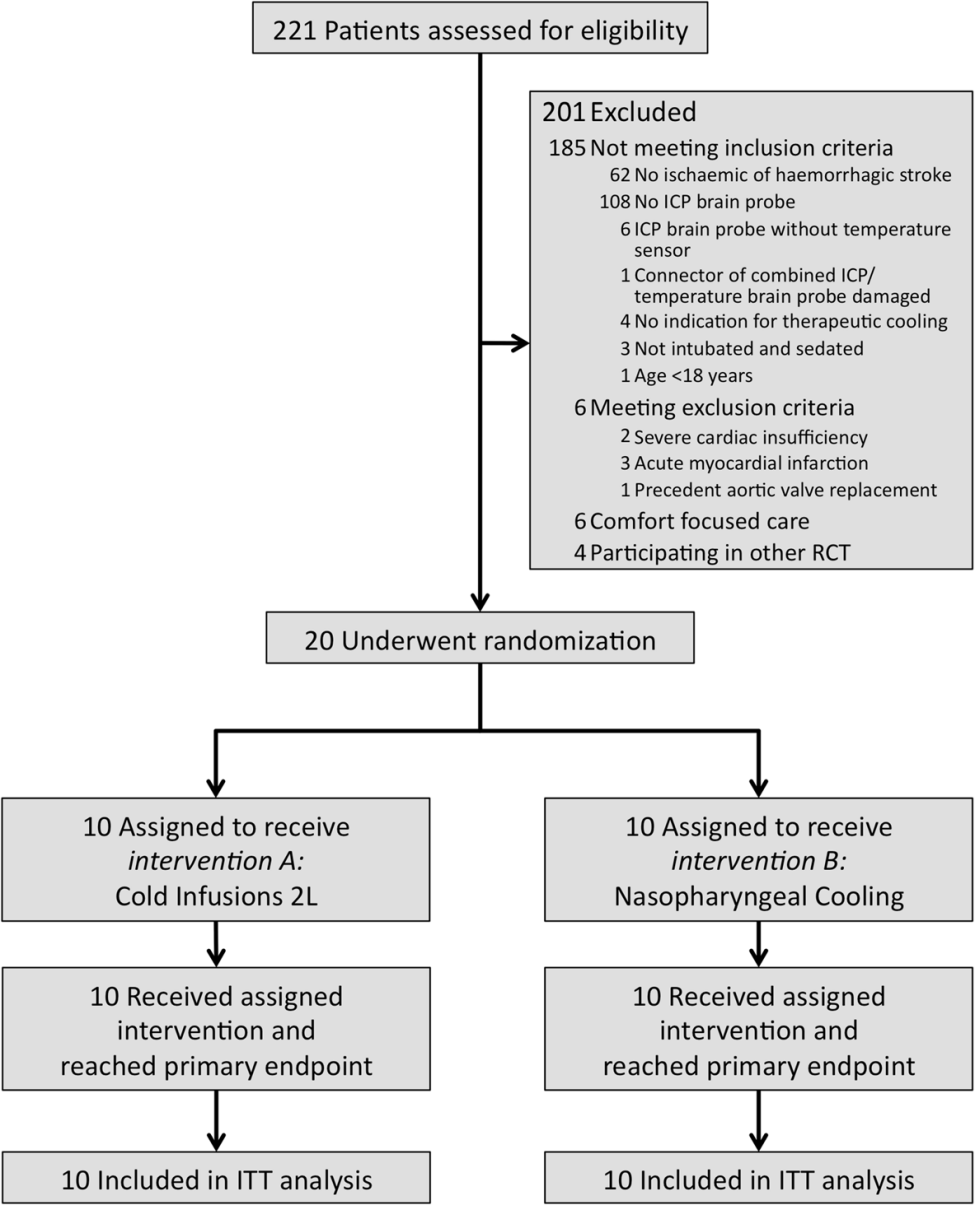
**Study flow chart.**

The study flow chart is shown in Figure 1. A total of 20 patients were enrolled in the study. Ten patients (seven ischaemic, three haemorrhagic stroke) received 2 L of CI in 33 ± 4 minutes and ten patients (four ischaemic, six haemorrhagic stroke) were treated with NPC for 60 minutes.

Baseline characteristics and outcome data are shown in Table 2.

**Table 2:** **Baseline characteristics and outcomes**

	**Cold infusion**	**Nasopharyngeal cooling**	***P*** **-value**
Patients; number	10	10	
Female; number (%)	4 (40%)	5 (50%)	n.s.^a^
Age, years; mean ± STD	65.4 ± 7.4	55.7 ± 11.5	0.038^b^
Weight, kg	83.5 ± 17.7	80.3 ± 12.7	n.s.^b^
Height, cm	173.1 ± 10.1	172 ± 10	n.s.^b^
Body mass index, kg/m^2^	27.9 ± 5.9	27.3 ± 2.4	n.s.^b^
Body surface area^d^, m^2^	2 ± 0.2	2 ± 0.2	n.s.^b^
Time from symptom onset to treatment, hours	69.4 ± 25.2	67.1 ± 31.9	n.s.^b^
Stroke type; number (%)			n.s.^a^
Ischaemic	7 (70%)	4 (40%)	
ICH	2 (20%)	5 (50%)	
SAH	1 (10%)	1 (10%)	
Scores			
Premorbid mRS; number (%)			n.s.^c^
0	7 (70%)	7 (70%)	
1	2 (20%)	2 (20%)	
2	1 (10%)	1 (10%)	
NIHSS admission; median (IQR)	14.5 (6.75-24.75)	26.5 (17.5-38)	n.s.^c^
mRS follow-up median (IQR)	4.5 (3.75-6)	4.5 (3–6)	n.s.^c^
Risk factors; number (%)			
Diabetes mellitus	0 (0%)	3 (30%)	n.s.^a^
Arterial hypertension	8 (80%)	8 (80%)	n.s.^a^
Hypercholesterolaemia	0 (0%)	1 (10%)	n.s.^a^
Atrial fibrillation	2 (20%)	0 (0%)	n.s.^a^

^a^Fisher's exact test; ^b^Student’s *t*-test; ^c^Mann–Whitney *U* test.; ^d^Mosteller formula. ICH, intracerebral haemorrhage; IQR, interquartile range; mRS, modified Rankin Scale; NIHSS, National Institutes of Health Stroke Scale; n.s. non-significant; SAH, subarachnoid haemorrhage; STD, standard deviation.

**Table 3:** **Intervention-related AE**

**ID**	**Intervention**	**Stroke type**	**Intervention-related AE**	**Subperiod**	**Treatment**
2	CI	Ischaemic	No	-	-
3	CI	Ischaemic	BSAS 3	II	Yes
4	CI	Ischaemic	∆SAP +42 mmHg, SAP 186 mmHg	III	No^a^
6	CI	ICH	∆SAP +33 mmHg	III	No
9	CI	ICH	∆SAP +43 mmHg	III	No
13	CI	Ischaemic	∆SAP +39 mmHg, ICP 27 mmHg	II	Yes
15	CI	Ischaemic	No	-	-
16	CI	Ischaemic	∆SAP +43 mmHg	III	No
17	CI	Ischaemic	∆SAP +28 mmHg	III	No
19	CI	SAH	∆SAP +18 mmHg, SAP 165 mmHg	IV	Yes
1	NPC	Ischaemic	∆SAP +35 mmHg	I	No
5	NPC	ICH	No	-	-
7	NPC	ICH	BSAS 3	I	Yes
8	NPC	SAH	∆SAP +14 mmHg, SAP 160 mmHg	I	Yes
10	NPC	Ischaemic	∆SAP +28 mmHg, SAP 187 mmHg	I	Yes
11	NPC	ICH	∆SAP +40 mmHg	I	No
12	NPC	Ischaemic	∆SAP +51 mmHg, ICP 27 mmHg	I	Yes
14	NPC	ICH	No	-	-
18	NPC	ICH	No	-	-
20	NPC	Ischaemic	∆SAP +25 mmHg	I	No

^a^SAP rose to 186 mmHg, but resolved spontaneously before treatment was initiated. Intervention-related AE of neurovital parameters for each patient, with the time of occurrence (subperiods I to IV), and whether or not the AE was treated. Treatment was indicated if SAP or ICP was higher than the predefined critical value (SAP ischaemic stroke ≥180 mmHg, haemorrhagic stroke ≥160 mmHg; ICP ≥20 mmHg), or the BSAS score was ≥1. ∆SAP indicates maximum delta SAP compared to baseline. BSAS, Bedside Shivering Assessment Scale; ICH, intracerebral haemorrhage; ICP, intracranial pressure; SAH, subarachnoid haemorrhage; SAP, systolic arterial pressure.

### Primary measurement: brain temperature

**Figure 2:**
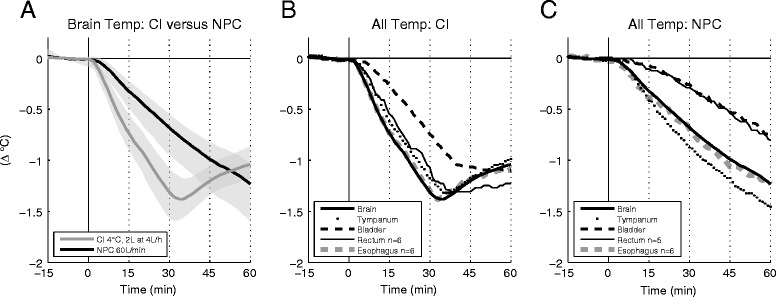
**Brain and body temperatures. A)** Mean brain temperature curves for CI (n = 10) versus NPC (n = 10) and corresponding 95% confidence intervals (grey shading). **B, C)** Mean curves of all body temperature measurements in comparison with brain temperature during treatment with CI **(B)** and NPC **(C)**. Sampling rate for all data acquisition was 1/minute.

Brain temperature was similar in the two study groups at baseline (CI 37.4 ± 0.4°C versus NPC 37.5 ± 1.1°C, n.s.), but differed significantly during the intervention (see Figure 2A): during the first 30 minutes of active treatment with CI (subperiods I and II) brain temperature decreased faster than during NPC (I: −0.31 ± 0.2 versus −0.12 ± 0.1°C, *P* = 0.008; II: −1.0 ± 0.3 versus −0.49 ± 0.3°C, *P* = 0.001, after 30 minutes: −1.31 ± 0.4 versus −0.68 ± 0.3°C, *P* < 0.001). In the CI group, after the infusion was discontinued, the intervention did not have a further decreasing effect on brain temperature, but brain temperature increased again after 3.5 ± 3.3 minutes. In the NPC group, after an initial increase, the brain cooling rate slightly decreased again during the course of active treatment (I: −0.12 ± 0.1°C, II: −0.37 ± 0.1°C, III: −0.34 ± 0.1°C, IV: −0.27 ± 0.1°C; after 60 minutes: −1.23 ± 0.4°C). All brain probes were inserted >3 cm below the cortical surface. In the NPC group the distance between the probe tip and the nasopharynx was 6.6 ± 1.7 cm. Only age differed significantly between the two groups (see Table 2) and body surface area (BSA) correlated with brain cooling during NPC (*r* = 0.6, *P* = 0.049). Considering that, we additionally performed analysis of covariance (ANCOVA) with age and BSA as covariates; this analysis yielded comparable results. All other influencing variables (including stroke type) listed in the supplementary statistics section [see Additional file 2] tested negative for correlation with brain cooling.

### Secondary measurements

#### Surrogate temperature sites

Data for surrogate temperature sites were available as follows: tympanum (20/20), bladder (20/20), rectum (CI 6/10, NPC 5/10), and oesophagus (CI 6/10, NPC 6/10). At baseline, body temperature at all sites was similar to brain temperature, with the exception of tympanic temperature, which was slightly lower (37.5 ± 0.7 versus 37.1 ± 0.7°C, *P* < 0.001). During both interventions oesophageal temperature correlated best with brain temperature (n.s. for difference; see Figure 2B), whereas decreases in bladder (*P* < 0.001) and rectal temperature (CI *P* = 0.011, NPC *P* <0.001) lagged distinctly in both groups. Tympanic temperature correlated well with brain temperature changes in patients receiving CI (n.s. for difference), but overestimated brain cooling in NPC-treated patients (*P* = 0.005).

### Neurovital parameters

**Figure 3:**
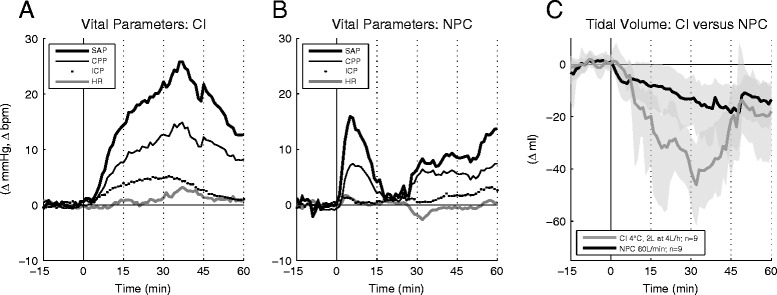
**Neurovital and respiratory parameters. A,** **B)** Mean curves of neurovital parameters during CI (*A*; n = 10), and NPC (*B*; n = 10). SAP, systolic arterial pressure; CPP, cerebral perfusion pressure; ICP, intracranial pressure; HR, heart rate. **C)** Mean inspiratory tidal volume curves for CI (n = 9) versus NPC (n = 9) and corresponding 95% confidence intervals (grey shading); respiratory data were not available for one patient in each interventional group. Sampling rate for all data acquisition was 1/minute.

Mean continuous curves of SAP, CPP, ICP and HR for each intervention are given in Figure 3. In the CI group, SAP, mean arterial pressure (MAP) and CPP were significantly higher than baseline in all subperiods (I to IV) and ICP was significantly increased during subperiods I to III (*P* <0.05 for all). During NPC, only SAP and MAP were significantly higher than baseline, and only in subperiod I. In both groups, the level of sedation (RASS −5) remained unchanged during the one-hour study period, and the changes of neurovital parameters did not correlate with the underlying stroke type and the sedation regimen.

Direct comparison of CI and NPC showed significantly higher values in the CI group in subperiod II for SAP, MAP, CPP and ICP (*P* <0.05). MAP and ICP were still significantly higher in subperiod III (*P* <0.05). HR and SpO_2_ saturation exhibited no significant differences between CI and NPC throughout the observation period.

CI provoked eleven intervention-related AEs of neurovital parameters during subperiods II to IV, of which three were classified as severe (two SAP, one shivering). In contrast, all eight NPC-associated AEs were confined to subperiod I. Four were classified as severe (three SAP, one ICP). Severe AEs were treated according to the local standard guidelines for management of BP, ICP and shivering. Intervention-related complications of individual patients are listed in Table 3.

### Respiratory parameters

Inspiratory tidal volume decreased during both interventions, although ventilator settings were kept stable. Changes were significant compared with baseline during subperiods II to IV in the CI group and during subperiods III and IV in the NPC group (*P* <0.05; see Figure 3C). In subperiods II and III, inspiratory tidal volume decreased to a greater extent in the CI than in the NPC group (*P* <0.05).

### Ventilator malfunction

Two cases of ventilator failure (SERVO-I) occurred during NPC – 56 minutes and 24 minutes after treatment start. In both cases, a change of the expiration cassette resolved the issue. Ventilation was bypassed with an Oxylog3000 (Draeger, Luebeck, Germany) for sixteen minutes and six minutes, respectively. The lowest SpO_2_ was 95% and 98%. We classified both events as possible intervention-related unanticipated serious adverse device effects (USADE).

### Chart and laboratory assessment

CI significantly affected volume balance compared with baseline and with the NPC group (CI 1,989 ± 190 ml versus NPC 0 ± 188 ml, *P* <0.001). No differences in laboratory values (including arterial blood gases) between the two treatment groups were found at any time. The supplementary table presents the detailed laboratory values [see Additional file 1].

### Imaging

Safety evaluation of brain imaging revealed no new bleeding events. In one patient the CT scan after NPC showed a retention of fluid perfluorohexane (PFH; 560 Hounsfield units) in the left sphenoid sinus [20]. This PFH retention had spontaneously resolved by the next CT examination two days later.

### Otorhinolaryngological evaluation

ORL evaluation on day 3 was available for 17 patients (CI 8/10, NPC 9/10). Overall, we observed four mild AEs without the necessity of further treatment, two in each interventional group. One patient in the CI group experienced minor bleeding at the larynx, which most likely occurred after tracheotomy was performed. In another patient of the CI group, ichor but no mucosal defect was found. One patient in the NPC group developed an epipharyngeal haematoma, and another suffered from swollen nasal conchae. These two AEs were classified as possibly intervention-related.

ORL evaluation at follow-up (>6 months) was available in 10 patients (of the remainder, six had died, while four declined examination). Rhinoscopic findings (CI five, NPC five) and the 16-item smell test with Sniffin’ Sticks (CI three, NPC five) were normal in all tested patients.

## Discussion

Induction methods for therapeutic cooling are underinvestigated [4]. For the first time, the iCOOL1 trial provides data on the effects of CI on brain temperature and neurovital parameters, demonstrates that oesophageal temperature is the best extracerebral surrogate for brain temperature during CI and NPC, and compares the effectiveness and safety of CI and NPC for induction of cooling in stroke patients.

### Brain cooling is faster during CI than during NPC

Subperiods I and II, the two subperiods during which both groups were actively treated, revealed faster brain cooling during CI (−1.31°C after 30 minutes) than during NPC (−0.68°C after 30 minutes). In the CI group, brain temperature reduction stopped only 3.5 minutes after cessation of CI, and brain temperature increased again (see Figure 2A). This is of great importance as it implies that additional methods for maintenance cooling should be commenced by the end of CI infusion in order to avoid fluctuations of brain temperature. Repetition of CI or infusion of higher volumes for bridging until maintenance cooling are most likely not an adequate solution owing to the high risk of harmful fluid overload [16]. On the other hand, it seems possible to stretch the duration of CI by decreasing the infusion rate. However, this would probably result in a loss of cooling efficiency. This is even more important, as the fast brain cooling with CI in our trial was obtained with a well-controlled CI temperature of 4°C, which cannot be guaranteed under all circumstances [26,27].

NPC yielded a continuous decrease throughout the 60 minutes of active treatment and reached −1.23°C at the end of subperiod IV. This finding is consistent with brain-cooling rates in patients who suffered traumatic brain injury or stroke, as previously described by Abou-Chebl *et al*. and our group [19,20]. In our observational period of one hour we captured the post-treatment period for the CI group, because an immediate increase of brain temperature after CI cessation occurred. However, since this was rather unexpected, this was not studied in the NPC group. As suggested by Abou-Chebl and colleagues we recorded the site of the brain temperature measurement (probe tip), as this may influence cooling rates during NPC [19]. The one-hour cooling rate (−1.2 versus -1.23°C) and the distance from the probe to the nasopharynx (6.6 versus 6.4 cm) were comparable in the two studies [20].

Yu and colleagues directly compared CI and NPC in a porcine model of prolonged cardiac arrest and reported that jugular vein temperature (measured as a surrogate for brain temperature) decreased faster in pigs treated with NPC [28]. This contrasts with our observation of faster brain cooling during CI. The closer anatomical proximity of the nasopharynx to a smaller brain and the existence of the rete mirabile in pigs relative to humans as well as the preserved cardiac circulation in stroke patients may explain these different findings. Considerations drawn from porcine cardiac arrest experiments measuring brain temperature, that NPC primarily cools the brain [29,30] are not supported by our results in stroke patients; in our study, oesophageal body core temperature decreased to the same degree as brain temperature.

### Bladder temperature is unsuitable as a surrogate for brain temperature during induction of cooling with CI or NPC

Very limited reports have been published on surrogate monitoring sites for brain temperature during rapid temperature changes [31,32]. Until now, data comparing brain temperature with temperatures in other parts of the body during CI have been completely lacking.

In this study, temperature changes in bladder and rectum, the most commonly used standard monitoring sites for body core temperature in the ICU, failed to promptly replicate brain temperature changes in both study groups (Figure 2B,C). This is in accordance with the results of Stone and colleagues, who investigated body temperatures in patients undergoing deep hypothermic circulatory arrest [32]. These authors also classified bladder and rectal temperature measurements as unsuitable for detecting rapid changes in brain temperature. Decreases in oesophageal temperature, on the other hand, were found to be accurate [32]. For the first time we can now confirm these findings for CI and NPC.

Our findings are of great importance: All but one [33] of the previously published large cardiac arrest/cooling induction trials that used CI [5,16,26] or NPC [17,18] monitored the slow-reacting bladder or rectal temperatures, or did not distinguish between core temperatures in different parts of the body but rather reported a composite core temperature. Furthermore, none of these studies monitored body temperature at the same site during cooling induction and maintenance cooling. It is, therefore, very unlikely that the fast increase of brain temperature after CI cessation would have been detected in those trials [34].

### Intervention-related AE of neurovital parameters in NPC-treated patients appear instantly, whereas onset is delayed in patients receiving CI

Our findings confirm our previous observation [20] that NPC may provoke immediate BP peaks even in deeply sedated patients and independent of stroke type (see Table 3). In our study, additional bolus medication reliably resolved these BP peaks. The BP increase we observed during CI seems to be directly linked to the administered volume of fluid and also did not correlate with the underlying stroke type. Tollofsrud *et al*. and Polderman *et al*. also found transient BP increases after CI in healthy volunteers and brain-injured patients [13,35]. In contrast, Kollmar and colleagues reported no BP increases in ten awake ischaemic stroke patients; however, they took measurements at 30-minute intervals, which limits comparability [11].

Importantly, even though both induction methods induced significant BP increases, brain imaging analysis showed no intracranial bleeding complications, also in those patients with haemorrhagic stroke. Nevertheless, our observation of blood pressure increases in both groups raises serious safety concerns especially when treating hyperacute haemorrhagic stroke.

In contrast to NPC, CI induced a more or less pronounced ICP increase in all patients, which, for the most part, can also be ascribed to the intravascular volume increase with a consecutive shift of intracranial compartments.

Furthermore, inspiratory tidal volume decreased to a noteworthy extent. Since patients remained deeply sedated (RASS −5) and ventilator settings of pc-CMV were not changed during the one-hour study period, an increase in respiratory resistance due to volume expansion [36] and cooling [37] may explain this finding.

We registered two cases of ventilator failure during NPC. Both malfunctions were unanticipated and serious (USADE) and are possibly intervention-related. In both cases, a change of the expiration cassette resolved the issue.

In summary, our findings of increased BP and ICP as well as other intervention-related and possibly intervention-related AEs raise concern regarding the safety of CI (4°C, 2 L at 4 L/hour) and NPC in stroke patients. Further study of safety is imperative before broader use.

### Limitations

Our study has some limitations. Group sizes in this randomised trial were rather small. However, a clearly defined endpoint using repeated-measurements ANOVA with a size of 10 in each group provided significant and robust results. The lack of a control group without cooling was inevitable, as inclusion was coupled to the study-independent indication for therapeutic cooling. We enrolled patients with different stroke entities (ischaemic stroke, ICH and SAH), but neither during CI nor during NPC brain cooling correlated with the underlying stroke type. Furthermore, both the frequency and the severity of adverse events were similar for the different stroke types and interventions (see Table 3). The requirement for brain temperature measurement inevitably leads to selection bias, as nearly all patients with an ICP/temperature brain probe are severely affected and all were intubated and sedated. Therefore, the findings of this study cannot be generalised to the very large majority of patients who are only mildly affected by stroke or who are treated in a prehospital setting. Fewer than 10% of patients with ischaemic stroke need intubation, a prerequisite for NPC [38]. As this trial was performed in one centre only, external validation of the results is necessary.

## Conclusions

In intubated stroke patients brain cooling is faster during active treatment with CI (4°C, 2 L at 4 L/hour) than during NPC. Importantly, contrary to previous expectations, brain cooling stopped soon after CI cessation. Our finding implies that additional methods for maintenance cooling should be commenced by the end of CI infusion in order to avoid fluctuations of brain temperature. NPC may be an alternative in intubated stroke patients when infusion of high volumes is contraindicated. Oesophageal, but neither bladder nor rectal temperature, is suited as surrogate for brain temperature during CI and NPC. Tympanic temperature seems feasible, too, during CI, when a certain offset in absolute values is kept in mind. Several severe adverse events in CI and in NPC demand further studying of safety.

## Key messages

- First study showing the effect of CI on brain temperature
- Brain cooling is faster during CI (4°C, 2 L at 4 L/hour) than during NPC.
- Brain temperature increases again soon after discontinuation of CI.
- Intervention-related AEs raise concern regarding the safety of CI (4°C, 2 L at 4 L/hour) and NPC in stroke patients.
- Oesophageal temperature is best suited as surrogate for brain temperature during both CI and NPC.

## Additional files

Additional file 1: Table S1. Safety laboratory. Mean values for each group and corresponding level of significance **P*<0.05. Additional file 2:
**Methods.**

## Abbreviations

AE: adverse effect
ANOVA: analysis of variance
BP: blood pressure
BSA: body surface area
BSAS: Bedside Shivering Assessment Scale
CI: cold infusion
CPP: cerebral perfusion pressure
CT: computed tomography
HR: heart rate
ICP: intracranial pressure
iCOOL1: Rapid Induction of COOLing in Stroke Patients trial
MAP: mean arterial pressure
NPC: nasopharyngeal cooling
ORL: otorhinolaryngological
pc-CMV: pressure-controlled continuous perfusion pressure
PFH: perfluorohexane
RASS: Richmond Agitation Sedation Scale
SAP: systolic arterial pressure
SpO_2_: oxygen saturation
