# Supplementary material for: Rapid Induction of COOLing in Stroke Patients (iCOOL1): a randomised pilot study comparing cold infusions with nasopharyngeal cooling
Source: Crit Care. 2014 Oct 27;18(5):582. doi: 10.1186/s13054-014-0582-1 (PMC4234831; doi:10.1186/s13054-014-0582-1)
Supplement: Additional file 1: Table S1. — Safety laboratory. Mean values for each group and corresponding level of significance *P<0.05. [file 13054_2014_582_MOESM1_ESM.pdf]

Additional Table: Safety laboratory. Mean values for each group and corresponding level of significance
** P<0.05*

	CI			RC			
	Mean	SD	†	Mean	SD		$P^{\dagger}$
**Inflammation**							
Leucocytes, /nL							
0h	11,92	4,34		11,02	4,10		n.s.
12h	11,05	3,69	*	13,51	10,85		n.s.
24h	9,78	3,16		12,84	7,75		n.s.
C-reactive protein, mg/l							
0h	104,92	64,37		85,25	53,37		n.s.
12h	118,75	51,74		132,26	44,65	*	n.s.
24h	129,93	53,25		168,26	42,23		n.s.
Procalcitonin, ng/ml							
0h	0,12	0,08		0,30	0,35		n.s.
12h	0,17	0,20		0,33	0,44		n.s.
24h	0,82	2,06		0,25	0,20		n.s.
**Electrolytes**							
Sodium, mmol/l							
0h	146,40	4,70		149,10	16,16		n.s.
12h	147,90	4,15		149,11	13,62		n.s.
24h	148,00	5,50		150,30	12,68		n.s.
Potassium, mmol/l							
0h	4,25	0,34		4,27	0,45		n.s.
12h	4,28	0,31		4,32	0,41		n.s.
24h	4,16	0,42		4,29	0,44		n.s.
**Brain injury**							
Neuron-specific enolase, µg/l							
0h	23,55	7,99		27,03	12,02		n.s.
12h	22,55	10,25		26,70	13,54		n.s.
24h	24,84	13,14		32,36	24,75		n.s.
S100, µg/l							
0h	0,65	0,58		0,88	0,98		n.s.
12h	0,70	0,68		1,41	1,85		n.s.
24h	0,53	0,38		1,26	1,76		n.s.
**Coagulation**							
International normalized ratio							n.s.
0h	1,03	0,08		1,02	0,07		n.s.
12h	1,03	0,09		1,03	0,07		n.s.
24h	1,01	0,09		1,05	0,08		
Thrombin time, sec							
0h	15,83	2,78		15,52	1,25		n.s.
12h	14,82	0,94		25,60	33,18		n.s.
24h	15,09	1,03		25,86	33,11		n.s.
Thrombocytes, /nl							
0h	215,10	40,54		237,30	35,72		n.s.
12h	212,70	36,55		218,60	42,24	*	n.s.
24h	202,20	31,25		203,00	47,90		n.s.
D-dimer, mg/l							

0h	3,25	2,25		5,76	4,92	*	n.s.
12h	3,40	3,36		3,67	3,11		n.s.
24h	3,94	4,37		4,76	6,90		n.s.
**Blood**							
Hemoglobin, g/dl							
0h	11,03	1,71		11,30	1,64		n.s.
12h	10,69	1,79		11,40	1,53		n.s.
24h	10,58	1,20		11,35	1,57		n.s.
Hematocrit, %							
0h	0,33	0,05		0,33	0,05		n.s.
12h	0,32	0,05	*	0,34	0,04		n.s.
24h	0,31	0,04		0,34	0,05		n.s.
**Cardiac markers**							
Creatin kinase, U/l							
0h	333,00	227,45		321,40	389,12		n.s.
12h	379,70	343,57		474,30	515,47		n.s.
24h	425,00	437,09		554,10	819,58		n.s.
CK-MB, U/l							
0h	16,40	6,57		14,60	6,95		n.s.
12h	16,50	7,37		21,80	15,91		n.s.
24h	17,70	9,55		30,40	25,61		n.s.
High-sensitive Troponin T, pg/ml							
0h	14,80	8,53		34,20	68,83		n.s.
12h	14,90	6,17		33,00	66,24	*	n.s.
24h	13,90	9,67		27,70	70,47		n.s.

**Blood gas analysis**							
Hemoglobin, g/dl							
0h	11,54	1,90		11,65	1,44		n.s.
2h	11,30	1,73		11,77	1,51		n.s.
Hämatocrit, %							
0h	0,34	0,05		0,35	0,05		n.s.
2h	0,33	0,04		0,35	0,04		n.s.
pH							
0h	7,43	0,05	*	7,39	0,05		n.s.
2h	7,38	0,04		7,37	0,05		n.s.
$PaCO_2$, mmHg							
0h	37,22	6,18		37,70	4,14		n.s.
2h	39,89	3,41		39,80	6,75		n.s.
PaO_2, mmHg							
0h	97,00	24,95		117,60	22,36		n.s.
2h	95,80	20,86		114,60	28,57		n.s.
Base excess, mmol/L							
0h	-0,48	2,35	*	-2,53	2,40		n.s.
2h	-2,15	1,97		-2,90	2,69		n.s.
Potassium, mmol/l							
0h	4,11	0,34		4,15	0,44		n.s.
2h	4,06	0,34		4,02	0,40		n.s.
Sodium, mmol/l							
0h	145,44	6,31		147,70	17,13		n.s.

2h	145,00	7,12		146,90	14,57	n.s.
Chlorid, mmol/L						
0h	115,90	6,21		114,11	5,58	n.s.
2h	117,10	6,47		118,60	14,25	n.s.
Glucose, mg/dl						
0h	144,20	14,90		133,60	28,23	n.s.
2h	136,30	20,33		134,40	36,65	n.s.
Lactate, mg/dl						
0h	11,73	5,36		9,93	3,35	n.s.
2h	9,42	1,26		10,64	5,42	n.s.
HCO_3, mmol/l						
0h	23,36	1,72	] *	22,52	2,05	n.s.
2h	22,14	1,55		21,96	2,52	n.s.
