# Supplementary material for: Rapid Induction of COOLing in Stroke Patients (iCOOL1): a randomised pilot study comparing cold infusions with nasopharyngeal cooling
Source: Crit Care. 2014 Oct 27;18(5):582. doi: 10.1186/s13054-014-0582-1 (PMC4234831; doi:10.1186/s13054-014-0582-1)
Supplement: Additional file 2: — Methods. [file 13054_2014_582_MOESM2_ESM.doc]

**Additional file 1: Methods**

*Medical equipment for multimodal neuromonitoring*

Brain, tympanic, bladder, rectal, and oesophageal temperature, all neurovital parameters (heart rate, arterial pressure, intracranial pressure (ICP), cerebral perfusion pressure and oxygen saturation) and ventilation parameters were monitored continuously on our standard monitoring system (Infinity Delta Monitor, Draeger, Telford, PA, USA). Brain temperature and ICP were measured with a combined ICP/temperature brain probe that was inserted >3cm below the cortical surface (Neurovent Temp or Neurovent PTO, accuracy ±0.1°C; Raumedic, Muenchberg, Germany). Tympanic, bladder, rectal, and lower oesophageal temperature were measured using temperature sensors of the 400 series Smiths Medical (accuracy ±0.2°C; Smiths Medical Rockland, MA, USA). Arterial pressure was measured via a radial or femoral artery catheter (Vygon, Aachen, Germany or Pulsion Medical Systems, Feldkirchen, Germany), and oxygen saturation was monitored with an adhesive digit SpO_2_ sensor (Nellcor, Covidien, Mansfield, MA, USA). The sampling rate for data acquisition was 1 per minute using Draeger software and the data were stored on a commercially available hard drive.

*Statistics*

Duration of baseline reading (15min), the division of the 1-hour study period into four subperiods (I to IV, 15min each) and statistical analyses were predefined in the study protocol. The start of the intervention (0min) was defined as the time at which cold infusions (CI) or the nasopharyngeal cooling (NPC) gas began to flow. ANOVA for repeated measurements was used to assess subperiod-dependent differences between the two interventions (primary endpoint), and intra-individual differences from baseline. To analyse the differences in dynamics of brain temperature and the neurovital parameters between the interventions, ANOVA with two dependent factors (CI and NPC) and time was used. To analyse the differences in dynamics between brain and other body temperatures within an intervention, ANOVA with two dependent factors (brain and body temperature) and time was used. Significant values were corrected according to Greenhouse and Geisser.

Pearson correlation was performed to analyse the influence of previously reported factors such as age, sex, body weight, body height, body mass index, body surface area (BSA; Mosteller formula), brain temperature at baseline, stroke type, time from stroke onset to cooling, distance between probe and palate, sedation regimen, and inflammatory response before application (C-reactive protein, leucocytes and procalcitonin) on brain cooling and on neurovital parameters.

Since age differed significantly between groups (see Table 2) and BSA correlated with brain cooling during NPC treatment, we additionally performed ANCOVA with age and BSA as covariates for primary endpoint analysis. *P* <0.05 was considered significant.

*References*

1. Abou-Chebl A, Sung G, Barbut D, Torbey M: **Local brain temperature reduction through intranasal cooling with the RhinoChill device: preliminary safety data in brain-injured patients**. *Stroke; a journal of cerebral circulation* 2011, **42**(8):2164-2169.

2. Lyden P, Ernstrom K, Cruz-Flores S, Gomes J, Grotta J, Mullin A, Rapp K, Raman R, Wijman C, Hemmen T: **Determinants of effective cooling during endovascular hypothermia**. *Neurocritical care* 2012, **16**(3):413-420.
